# Quantification of Photosynthetic Pigments in *Neopyropia yezoensis* Using Hyperspectral Imagery

**DOI:** 10.34133/plantphenomics.0012

**Published:** 2023-01-10

**Authors:** Shuai Che, Guoying Du, Xuefeng Zhong, Zhaolan Mo, Zhendong Wang, Yunxiang Mao

**Affiliations:** ^1^ Key Laboratory of Marine Genetics and Breeding (Ministry of Education), College of Marine Life Sciences, Ocean University of China, Qingdao, 266003, China.; ^2^ Key Laboratory of Utilization and Conservation of Tropical Marine Bioresource (Ministry of Education), College of Fisheries and Life Science, Hainan Tropical Ocean University, Sanya, 572002, China.; ^3^ Yazhou Bay Innovation Institute, Hainan Tropical Ocean University, Sanya, 572025, China.; ^4^ Laboratory for Marine Biology and Biotechnology, Pilot National Laboratory for Marine Science and Technology (Qingdao), Qingdao, 266073, China.

## Abstract

Phycobilisomes and chlorophyll-a (*Chla*) play important roles in the photosynthetic physiology of red macroalgae and serve as the primary light-harvesting antennae and reaction center for photosystem II. *Neopyropia* is an economically important red macroalga widely cultivated in East Asian countries. The contents and ratios of 3 main phycobiliproteins and *Chla* are visible traits to evaluate its commercial quality. The traditional analytical methods used for measuring these components have several limitations. Therefore, a high-throughput, nondestructive, optical method based on hyperspectral imaging technology was developed for phenotyping the pigments phycoerythrin (PE), phycocyanin (PC), allophycocyanin (APC), and *Chla* in *Neopyropia* thalli in this study. The average spectra from the region of interest were collected at wavelengths ranging from 400 to 1000 nm using a hyperspectral camera. Following different preprocessing methods, 2 machine learning methods, partial least squares regression (PLSR) and support vector machine regression (SVR), were performed to establish the best prediction models for PE, PC, APC, and *Chla* contents. The prediction results showed that the PLSR model performed the best for PE (*R*_Test_^2^ = 0.96, MAPE = 8.31%, RPD = 5.21) and the SVR model performed the best for PC (*R*_Test_^2^ = 0.94, MAPE = 7.18%, RPD = 4.16) and APC (*R*_Test_^2^ = 0.84, MAPE = 18.25%, RPD = 2.53). Two models (PLSR and SVR) performed almost the same for *Chla* (PLSR: *R*_Test_^2^ = 0.92, MAPE = 12.77%, RPD = 3.61; SVR: *R*_Test_^2^ = 0.93, MAPE = 13.51%, RPD =3.60). Further validation of the optimal models was performed using field-collected samples, and the result demonstrated satisfactory robustness and accuracy. The distribution of PE, PC, APC, and *Chla* contents within a thallus was visualized according to the optimal prediction models. The results showed that hyperspectral imaging technology was effective for fast, accurate, and noninvasive phenotyping of the PE, PC, APC, and *Chla* contents of *Neopyropia* in situ. This could benefit the efficiency of macroalgae breeding, phenomics research, and other related applications.

## Introduction

Red macroalgae (Rhodophyta) are a group of ancient photosynthetic eukaryotes distributed worldwide [1,2]. Phycobilisomes and chlorophyll-a (*Chla*) are the main photosynthetic pigments of red algae. Phycobilisomes play essential roles in red algae, serving as the primary light-harvesting antennae for photosystem II, and can transfer energy to photosystem I [3,4]. Phycobilisomes are considered an indicator of total protein content because they make up ~40% of cell proteins [5]. As large protein complexes, phycobilisomes consist of phycobiliproteins including phycoerythrin (PE), phycocyanin (PC), allophycocyanin (APC), and linker proteins. *Chla* is a critical pigment in all photosynthetic organisms, and its content is commonly used to estimate the N content in higher plants [6].

*Neopyropia*, also known as nori or laver, is an economically important red macroalga widely farmed in East Asian countries, including China, Japan, and Korea [7]. The global production of *Neopyropia* was almost 3 million tons (fresh weight) and was valued at approximately US$2.66 billion in 2019 [7]. *Neopyropia* spp. thalli have a delicious flavor and contain numerous nutritional components. In particular, it has a high protein content of ~25 to 40% dry weight, which is even higher than that of soybean [8–10]. For *Neopyropia*, the contents and ratios of 3 main phycobiliproteins (PE, scarlet color; PC, blue color; APC, indigo-blue color) and *Chla* (green) not only contribute to the total protein content but also determine the color of the blade and is a visible trait to evaluate its commercial quality [8,11,12].

The traditional analytical methods used for measuring phycobiliprotein and chlorophyll contents include ultraviolet-visible (UV-Vis) spectrophotometry [13], high-performance liquid chromatography [14], and liquid chromatography–mass spectrometry [15]. Although stable and accurate, these conventional methods are costly, laborious, time-consuming, destructive, highly experience-dependent, cannot be performed on a large scale, and cannot satisfy the requirements of high-throughput and nondestructive phenotyping [16]. Thus, rapid and efficient approaches for evaluating phenotraits must be developed.

In recent decades, phenomics has been indicated as an important tool to address and understand causal links between genotypes and environmental factors and phenotypes [17,18]. However, the lack of fast, high-throughput, nondestructive, and accurate phenotyping method is limiting the efficiency of phenomics research and modern plant breeding [19,20]. With the development of sensors and technology, hyperspectral imaging (HSI) is a promising method for solving this bottleneck [6,21,22]. As an integration of imaging and conventional spectroscopy, HSI can generate data in the form of a 3-dimensional (3D) spatial map of spectral variation, with the first 2 dimensions providing spatial information and the third accounting for spectral information [23]. With this combination, the HSI system can simultaneously extract spatial and spectral information related to plants’ structural texture and physiology [24,25]. In general, HSI systems record a wide range of reflectance spectra, including visible (Vis; 400 to 700 nm), near-infrared (NIR; 700 to 1000 nm), and shortwave infrared (SWIR; 1100 to 2500 nm) ranges. The reflectance’s strength and wavelength depend on the nature of biological materials [26]. Typically, for terrestrial plants, the Vis reflectance is mainly influenced by photosynthetic pigments, such as chlorophyll, carotenoids, and anthocyanins; the NIR reflectance is determined by light scattering by the tissue structure; and the SWIR reflectance is dominated by dry matter and water [23,27,28]. However, HSI is usually influenced by external factors, imaging components, and sample complexities. Spectral preprocessing methods are commonly used before applying any modeling tools to reduce the variations and obtain more homogeneous and less noisy data from HSI [26]. Multivariate analysis methods based on chemometrics or machine learning methods, such as partial least squares regression (PLSR), support vector machine regression (SVR), random forest, and convolutional neural networks, provide robust models for plant biochemical data and spectral information [29–31].

Numerous studies based on HSI systems have been used to accurately assess crop traits in a high-throughput manner [32–34]. As traits can be accurately measured across hundreds of individuals of a target species, quantitative genetic tools can be used to identify regions of the genome or specific genes controlling variation in the target trait [34]. Herzig et al. [35] applied HSI to estimate the concentrations of 15 grain elements in 1,420 barley lines and located 75 quantitative trait loci using a genome-wide association study (GWAS). Ikeogu et al. [36] predicted carotenoids in 173 cassava root samples using HSI combined with the random forest method. Using the developed models, GWAS was conducted to analyze the substantial genomic regions associated with carotenoids using 594 cassava clones. Sun et al. [37] used HSI combined with GWAS to dissect the protein content of rice seeds and identified 65 genes. Barnaby et al. [38] evaluated rice grain quality using high-throughput HSI for GWAS and revealed plausible candidate genes. Comparatively, algae have been studied less in high-throughput phenotyping research than terrestrial plants [39,40]. Most studies quantify algal biochemical contents using non-imaging optical spectroscopy, which provides information on a limited surface area [41–44]. The literature on the use of HSI technology to quantitatively measure the biochemical properties of algae is scant. Vahtmäe et al. [45] predicted the *Chla + Chlb* concentrations in the brown alga *Fucus vesiculosus* and the green alga *Cladophora glomerata* and *Ulva intestinalis* using HySpex (Norsk Elektro Optikk, Norway) with a spectral range from 410 to 988 nm. The lipid concentration and fatty acid unsaturation of the green microalgae *Scenedesmus obliquus* were investigated using HSI [46,47]. Nevertheless, to our knowledge, no such study has been conducted on red macroalgae.

In this study, experiments were conducted to evaluate the potential of HSI for determining phycobiliproteins (PE, PC, and APC) and *Chla* content in red macroalgae *Neopyropia yezoensis*. This was done to establish a rapid, high-throughput, and nondestructive method to displace traditional time-consuming and destructive methods for macroalgal phenotyping. Two types of multivariate modeling analysis methods based on PLSR and SVR were evaluated to establish optimal prediction models.

## Materials and Methods

### Algal materials

The genetically pure line PYL201306-440 (RZ) of *N. yezoensis* cultured in the laboratory was used in this study. Thalli were cultured in sterilized natural seawater with Provasoli’s enrichment solution (PES) medium at 10 °C, with a light concentration of 60 μmol photons m^−2^ s^−1^ and a 12-h light:12-h dark photoperiod. The culture medium was renewed every 3 d until the thalli reached 5 cm in length. The thalli were then transferred to other containers to perform experimental incubation.

The *N. yezoensis* thalli were treated under different levels of nutrients and different light qualities to obtain different pigment concentrations. This was based on previous studies that indicated that nutrient limitation and different light qualities would change the concentration and composition of phycobiliprotein and *Chla* in red algae [48,49].

The thalli were subjected to 3 different nutrient levels (low, normal, and high) at 10 °C, with a light concentration of 60 μmol photons m^−2^ s^−1^ and a 12-h light:12-h dark photoperiod. Artificial seawater [50] without nitrogen and phosphorus supply was used for the low-nutrient level treatment and sterilized seawater with 1× PES and 3× PES for normal and high-nutrient levels, respectively. The culture medium was changed daily to ensure an adequate nutrient supply. The experiment was conducted over 9 d. Three thalli were collected from each nutrient level from the second day and denoted as one sample. Three replicate samples for each nutrient level were collected daily for further analysis.

An MC 1000 test tube photobioreactor (PSI Photon Systems Instruments, Drasov, Czech Republic) was used to cultivate the thalli at different light qualities. The thalli were subjected to 8 light qualities with a light concentration of 60 μmol photons m^−2^ s^−1^ and a 12-h light:12-h dark photoperiod at 10 °C. The thalli were cultured in sterilized natural seawater with 1× PES medium, which was changed daily to ensure an adequate nutrient supply. Three thalli were collected from each light quality after 7 d of cultivation in the bioreactor and denoted as one sample. Three replicate samples of each light quality were collected for further analysis.

### Hyperspectral image acquisition

**Fig. 1.**
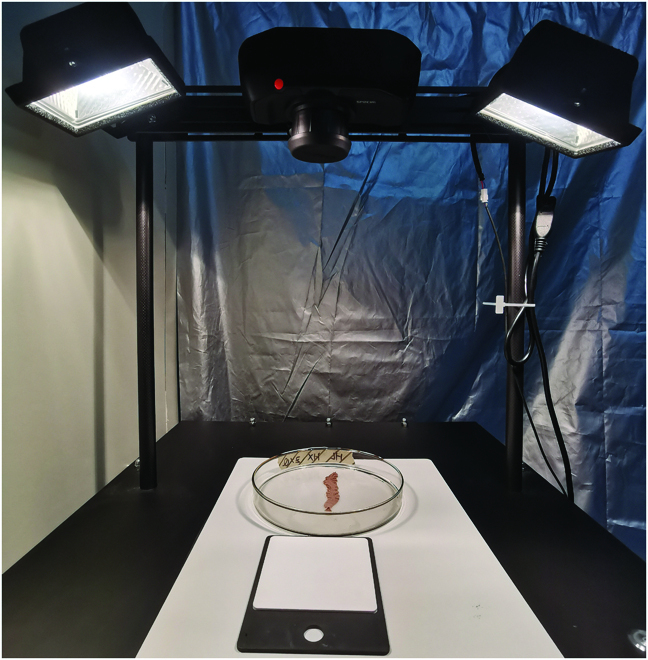
The interior view of the hyperspectral platform.

An HSI system that mainly consisted of a hyperspectral camera (Specim IQ, Specim Ltd., Oulu, Finland), two 150-W halogen lamps, and a piece of 70% reflection Teflon sheet (Jingyi Optoelectronics Technology Co. Ltd., Guangzhou, China) was designed to obtain the hyperspectral image data (Fig. 1). The spectral measurements were obtained with a line scanner and comprised a wavelength range of 400 to 1000 nm (Vis–NIR) with a 7-nm spectral resolution and 204 spectral bands in total. The image size was 512 × 512 pixels [51]. Two halogen lamps were placed on the sides, pointing to the camera’s viewing area. During the acquisition of the hyperspectral images, the thalli were spread on a glass plate on a Teflon sheet and brought into the camera’s viewing area with a horizontal white reference panel (99% light reflection) beside the sample. In the present study, the lens-to-sample distance was approximately 40 cm, and the exposure time was 8 ms. The camera recorded the dark reference automatically, whereas the white reference was recorded simultaneously with the sample. Image correction was performed automatically using the software loaded into the equipment. The image data were processed and stored in the default recording mode. The study acquired hyperspectral images of 96 laboratory samples for further analysis.

### Image segmentation and spectral data extraction

After the conversion of raw spectral radiance images to spectral reflectance images, the average reflectance of the region of interest (ROI) for each sample was extracted using the Environment for Visualizing Images (ENVI) 5.1 software (Exiles Visual Information Solutions, USA). When selecting ROIs, shadows and highlights in the image were excluded.

### Spectral data preprocessing

Radiometric calibration using a white reference was performed to compensate for the effect of horizontal in-plane inhomogeneous illumination [27]. However, the hyperspectral image collection process is usually influenced by many factors [52]. The preprocessing methods are valid tools for highlighting the desired spectral characteristics and for reducing the data variability before modeling [29,53]. In this study, 5 preprocessing methods, i.e., Savitzky–Golay (S-G, polynomial order: 2; points of window: 5) smoothing method + standardization, S-G + standard normal variate (SNV), S-G + first derivative, S-G + second derivative, and multiplicative scatter correction (MSC), were performed prior to building prediction models to improve the performance.

### Extraction and determination of the pigments

Phycobiliproteins and *Chla* extractions were performed immediately after hyperspectral data acquisition to obtain the pigment concentration of each sample. Each fresh sample was first weighed and then frozen in liquid nitrogen for grinding using a tissue lyser (TissueLyser-24L, Jingxin Industrial Development Co. Ltd, Shanghai, China) after removing all surface moisture. Next, 4 ml of precooled phosphate-buffered saline (50 mM, pH 6.8) was added. The homogenized sample was maintained in an ice bath in the dark and frequently vibrated for 1 h. The sample was then centrifuged at 13,000 × *g* and 4 °C for 20 min. The sample supernatant was transferred to a new tube and maintained in an ice bath under dark conditions. Then, 4 ml of precooled phosphate-buffered saline (50 mM, pH 6.8) was added to the precipitate for repeated extraction. The mixture of the 2 supernatants was treated as the initial crude phycobiliprotein extract of each sample. Subsequently, 6 ml of 80% (v/v) acetone was added to the precipitate, and the sample was incubated in the dark for 20 min with continuous oscillation and then centrifuged at 10,000 × *g* and 4 °C for 10 min to obtain the crude chlorophyll extracts. This process was performed in an ice bath. For each sample, the measurements were performed in triplicate. The absorbance of the phycobiliprotein was measured at 498, 614, and 651 nm, and the PE, PC, and APC contents (mg/g) were calculated according to the equations described by Kursar et al. [54]. The absorbance of *Chla* was measured at 646 and 663 nm. The *Chla* content (mg/g) was calculated according to the equation described by Wellburn [55].

### Multivariate modeling analysis

PLSR and SVR were used to predict phycobiliprotein and *Chla* content (mg/g) in the thalli of *N. yezoensis* using spectral information. The 96 laboratory samples were split into 2 groups: 79 (approximately 80%) for model training and 17 (approximately 20%) for testing.

PLSR is one of the most commonly used methods for hyperspectral image data analysis [53]. It is used to optimize the covariance between the label and linear combinations of features by simultaneously decomposing the multivariate input data [56]. A leave-one-out cross-validation scheme was employed to calibrate the model. Fifty latent factors were considered for the PLSR model establishment, and the size of the models was determined by the number of latent variables (*n*_LV_), representing the minimum root mean square error of cross-validation (RMSECV).

SVR is a nonlinear machine learning method that can efficiently perform nonlinear regression using a kernel trick [57]. SVR is appealing in the spectral regression field because of its ability to handle small training datasets successfully [58]. A radial basis function kernel with a Gaussian profile was used to reduce the computational complexity of the SVR training procedure. Regularization parameters C and g were optimized to improve the performance of the SVR model. C controls the trade-off between minimizing model complexity and training error, and g is the width of the radial basis function [41]. The performance of the SVR model depends on the advisable choice of the 2 parameters to determine a hyperplane with the minimum predictive error.

### Model performance evaluation

The model performance was statistically evaluated using the coefficient of determination (*R*^2^), RMSE, ratio of performance to deviation (RPD), and mean absolute percent error (MAPE). The RPD is a widely used criterion for predictive model evaluation in chemometrics modeling. Higher RPD values correspond to better analytical performance [59,60]. In this study, we divided the models into 3 groups according to different RPD values based on previous studies. The model with RPD > 3.0 means excellent quantitative prediction with high confidence. The model with an RPD between 2.0 and 3.0 means a good quantitative prediction that may not be used for quantitative analysis but could be used for qualitative analysis. If RPD < 2.0, then the model may not be useful and should be improved further. The MAPE value was used to assess the accuracy of the model prediction. If the value was <15%, then the model had high accuracy. The values of these indices were calculated using the following equations:

$$\text{RMSE}=\sqrt{\frac{1}{N}\times \sum {\left(y_{i}-{\hat{y}}_{i}\right)}^{2}}$$

RPD=SD/RMSE

$$\text{MAPE}=\frac{\frac{1}{N}\sum \left|y_{i}-{\hat{y}}_{i}\right|}{\text{mean}}\times 100\%$$

where *N* is the number of *N. yezoensis* in the training set (79) or test set (17); *y_i_* and ${\hat{y}}_{i}$ are the laboratory-measured and model-predicted values, respectively; and SD and mean are the standard deviation and mean of the laboratory-measured values.

MATLAB 2019b (MathWorks Inc., Natick, USA) was used for data analysis including spectral preprocessing, PLSR, and SVR modeling.

### Validation of the optimal models using field samples

Twenty-four field samples collected from the Qingdao coast were used for further validation of optimal models. The hyperspectral images were acquired under the same conditions as the laboratory samples, and pigment extraction was performed as described above. The *R*_W_^2^, RMSE, MAPE, and residuals were used to evaluate the model performance for the field samples.

### Prediction maps of phycobiliproteins and *Chla* contents in *N. yezoensis*

The best models for the 4 pigments were selected for the prediction at the individual pixel level. Prediction maps are beneficial for quantitative observation of phycobiliproteins and *Chla* at any point in the thalli. The 4 pigments were visualized using MATLAB 2019b.

## Results

### Spectral characteristics of *N. yezoensis*

**Fig. 2.**
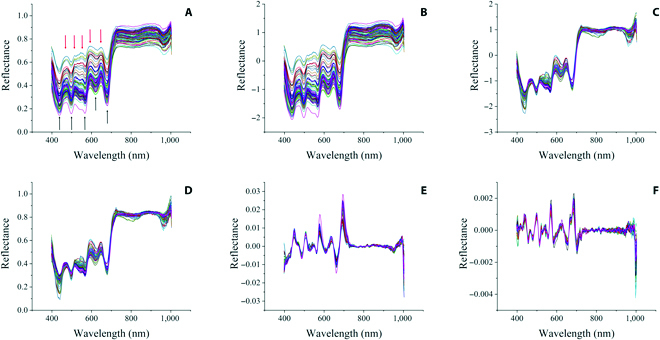
The raw average reflectance (A), Savitzky–Golay (S-G) smoothing method + standardization preprocessing (B), S-G + standard normal variate (SNV) preprocessing (C), multiplicative scatter correction (MSC) preprocessing (D), S-G + first derivative preprocessing (E), and S-G + second derivative preprocessing (F) reflectance of *N. yezoensis* samples in Vis–NIR ranges.

The mean reflectance spectra of the ROI were extracted from 96 hyperspectral images to develop prediction models. Figure 2A shows the raw average spectra of the *N. yezoensis* samples under different treatments in the Vis–NIR range. Overall, consistent spectral patterns were observed for different treatments in the 400- to 1000-nm wavelength region. The typical absorption wavelengths of *N. yezoensis* in the Vis spectral region were 440, 500, 570, 620, and 660 nm (Fig. 2A). There were also 5 typical reflectance peaks at approximately 475, 522, 557, 595, and 651 nm (Fig. 2A). A dramatic increase in reflectance was observed at the transition from the Vis to the NIR wavelength and maintained high values throughout the NIR domain. Apparent differences in reflectance values were observed in the Vis–NIR spectral region among the samples.

There were also overlaps and fluctuations in the original spectra, which adversely affected the model performance. To minimize the impact of interference and achieve an accurate and reliable model for prediction, 5 preprocessing combination methods, i.e., S-G smoothing + standardization (Fig. 2B), S-G + SNV (Fig. 2C), MSC (Fig. 2D), S-G + first derivative preprocessing (Fig. 2E), and S-G + second derivative preprocessing (Fig. 2F), were performed before model generation.

### Reference data of phycobiliproteins and *Chla* contents in *N. yezoensis*

It is essential to ensure an adequate range and precision of trait data as a reference for developing spectral calibrations and modeling. In this study, fresh *N. yezoensis* thalli treated with different levels of nutrients and light qualities and collected from the field contained varied phycobiliprotein and chlorophyll contents. The descriptive statistics for the PE, PC, APC, and *Chla* content of the samples are summarized in Table 1.

**Table 1.** Reference data of phycobiliproteins (PE, PC, and APC) and *Chla* contents by UV-Vis spectrophotometry method.

Constituent	Samples	Range (mg/g)	Mean (mg/g)	Standard deviation (mg/g)
PE	Lab cultured	1.840–13.450	6.790	2.427
	Field-collected	0.939–6.229	2.643	1.467
PC	Lab cultured	0.960–5.270	3.920	1.107
	Field-collected	0.589–4.650	2.046	1.186
APC	Lab cultured	0.430–2.350	1.440	0.481
	Field-collected	0.294–1.838	0.901	0.429
*Chla*	Lab cultured	0.290–2.230	1.360	0.498
	Field-collected	0.299–0.845	0.524	0.174

### Multivariate modeling analysis by PLSR and SVR

#### PLSR models based on full spectra (400 to 1000 nm)

Linear PLSR models were performed based on full spectra (400 to 1000 nm) with preprocessing methods to predict the content of PE, PC, APC, and *Chla* in *N. yezoensis* thalli. As listed in Table 2, the 5 preprocessing methods resulted in diverse prediction performances. Among them, the model established with S-G smoothing followed by standardization could predict all 4 pigments optimally, especially PE, which was more predictable than the others in the Vis–NIR ranges. With this combination, the optimal number of latent variables (*n*_LV_) for PE was 10, with the highest training *R*^2^ (*R*_Train_^2^) value of 0.92 and test *R*^2^ (*R*_Test_^2^) value of 0.96. The RMSE_Train_ and RMSE_Test_ values of PE content prediction were 0.6614 and 0.4804 mg/g for the training and test sets, respectively. For PC and *Chla*, the prediction models were satisfactory, with the *R*_Test_^2^ above 0.9. However, for APC prediction, the *R*^2^ values were low at 0.74 and 0.79, with an RMSE of 0.2369 and 0.2520 mg/g for the training and test datasets, respectively. In this study, MAPE values for the best model of PE, PC, and *Chla* were 8.31%, 9.94%, and 12.77%, respectively. However, the MAPE value of APC was 21.03%, which meant lower accuracy. The PE, PC, and *Chla* models developed in this study using the PLSR method had RPD values of more than 3.0, which could be used for quantitative evaluation; however, the APC model could only be used for qualitative analysis.

**Table 2.** Training and test results of estimating phycobiliproteins (PE, PC, and APC) and *Chla* contents of *N. yezoensis* using the PLSR method (the optimal models are in bold typeface).

Pigment	Preprocess method	Training			Test			
		*R*_Train_^2^	RMSE_Train_(mg/g)	*n*_LV_	*R*_Test_^2^	RMSE_Test_ (mg/g)	MAPE (%)	RPD
PE	**S-G + standardization**	**0.92**	**0.6614**	**10**	**0.96**	**0.4804**	**8.31**	**5.21**
	S-G + SNV	0.91	0.7246	10	0.95	0.5992	8.36	4.18
	S-G + first derivative	0.90	0.7397	7	0.94	0.5928	11.28	4.23
	S-G + second derivative	0.88	0.8317	5	0.88	0.8573	16.93	2.92
	MSC	0.90	0.7451	10	0.95	0.5925	9.50	4.23
PC	**S-G + standardization**	**0.91**	**0.3258**	**8**	**0.93**	**0.3347**	**9.94**	**3.82**
	S-G + SNV	0.89	0.3585	7	0.91	0.4273	13.42	2.99
	S-G + first derivative	0.87	0.3876	6	0.94	0.3695	11.38	3.55
	S-G + second derivative	0.80	0.4805	13	0.84	0.6238	17.43	2.04
	MSC	0.89	0.3629	7	0.91	0.4212	13.28	3.03
APC	**S-G + standardization**	**0.74**	**0.2369**	**9**	**0.79**	**0.2520**	**21.03**	**2.24**
	S-G + SNV	0.72	0.2496	7	0.77	0.2704	23.51	2.08
	S-G + first derivative	0.72	0.2467	5	0.67	0.3139	28.25	1.79
	S-G + second derivative	0.65	0.2749	5	0.67	0.3181	27.96	1.77
	MSC	0.72	0.2486	7	0.74	0.2923	25.65	1.93
*Chla*	**S-G + standardization**	**0.82**	**0.2049**	**9**	**0.92**	**0.1530**	**12.77**	**3.61**
	S-G + SNV	0.79	0.2236	9	0.90	0.1885	17.05	2.93
	S-G + first derivative	0.78	0.2309	5	0.83	0.2306	23.01	2.39
	S-G + second derivative	0.71	0.2678	5	0.83	0.2278	22.14	2.42
	MSC	0.78	0.2300	9	0.89	0.1968	18.68	2.81

#### SVR models based on full spectra (400 to 1000 nm)

The same training and test datasets as those of the PLSR model were used to construct the SVR model. The PE content was best predicted by the SVR model combining MSC preprocessing, and the *R*_Test_^2^, RPD, and MAPE_T_ were 0.96%, 4.50%, and 9.08%, respectively (Table 3). The PC content was well predicted by the SVR model with S-G + SNV processing and the *Chla* content by SVR with S-G + first derivative. All PE, PC, and *Chla* contents were quantitatively predicted, as indicated by RPD > 3.0. The SVR models for APC were still insufficient for quantitative prediction, with an optimal RPD of 2.53. Compared with the PLSR models, the best prediction models of the 4 pigments in SVR modeling showed a slight difference in model performance. The optimal SVR model of PE using the preprocessing method of MSC showed less accuracy than the optimal PLSR model of PE using S-G + standardization preprocessing. However, for PC and APC, the optimal SVR models combining S-G + SNV preprocessing showed better performance than PLSR models combining S-G + standardization preprocessing with *R*_Test_^2^ improved from 0.93 to 0.94 and from 0.79 to 0.84, respectively. The RMSE_Test_ values of SVR optimal models for PC and APC were reduced to 0.3068 and 0.2232 mg/g, corresponding to the improvements in the prediction accuracy with MAPE values of 7.18% and 18.25%, respectively. Meanwhile, the RPD values of PC and APC were further improved to 4.16 and 2.53. The performance of the *Chla* prediction models constructed by PLSR and SVR showed almost the same results, indicating that the linear and nonlinear regression methods had no significant difference for *Chla* content prediction.

**Table 3.** Training and test results of estimating phycobiliproteins (PE, PC, and APC) and *Chla* contents of *N. yezoensis* using the SVR method (the optimal models are in bold typeface).

Pigment	Preprocess method	Training			Test			
		C	g	*R*_Train_^2^	*R*_Test_^2^	RMSE_Test_	MAPE_T_ (%)	RPD
PE	S-G + standardization	2097152	0.0000038147	0.98	0.94	0.6222	11.72	4.03
	S-G + SNV	512	0.0039063	0.98	0.94	0.6663	11.38	3.76
	S-G + first derivative	16	0.0078125	0.97	0.95	0.6014	10.88	4.17
	S-G + second derivative	32	0.0078125	0.99	0.92	0.7540	12.44	3.32
	**MSC**	**128**	**0.0078125**	**0.98**	**0.96**	**0.5567**	**9.08**	**4.50**
PC	S-G + standardization	2097152	0.0000038147	0.97	0.93	0.3726	8.03	3.43
	**S-G + SNV**	**8**	**0.03125**	**0.97**	**0.94**	**0.3068**	**7.18**	**4.16**
	S-G + first derivative	1048576	0.00000011921	0.95	0.90	0.4310	12.78	2.96
	S-G + second derivative	4	0.0625	0.99	0.92	0.3900	10.71	3.27
	MSC	256	0.0019531	0.96	0.94	0.3372	10.17	3.79
APC	S-G + standardization	8	0.125	0.97	0.75	0.2914	25.48	1.94
	**S-G + SNV**	**8**	**0.125**	**0.97**	**0.84**	**0.2232**	**18.25**	**2.53**
	S-G + first derivative	4	0.03125	0.96	0.75	0.2903	25.04	1.94
	S-G + second derivative	524288	0.00000011921	0.91	0.57	0.3584	29.78	1.57
	MSC	8	0.25	0.97	0.84	0.2345	19.97	2.41
*Chla*	S-G + standardization	16	0.125	0.97	0.84	0.2208	19.23	2.50
	S-G + SNV	128	0.03125	0.97	0.86	0.260	16.36	2.45
	**S-G + first derivative**	**2**	**0.03125**	**0.97**	**0.93**	**0.1537**	**13.51**	**3.60**
	S-G + second derivative	4	0.03125	0.97	0.89	0.1958	17.28	2.82
	MSC	512	0.0039063	0.96	0.88	0.2342	22.25	2.36

**Fig. 3.**
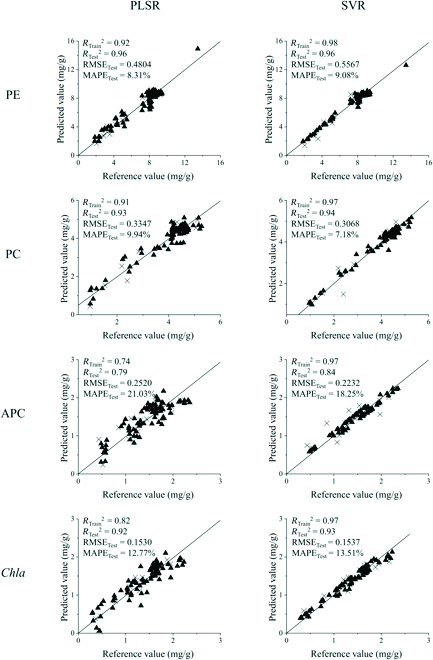
Scatter diagrams of the reference value vs. the predicted value of PE, PC, APC, and *Chla* contents by optimal PLSR and SVR models. The training sets are denoted by ▲ and the test sets by ×.

The dispersions between the reference and predicted values of PE, PC, APC, and *Chla* in the optimal integrated PLSR and SVR models (bold in Tables 2 and 3) are shown in Fig. 3. In conclusion, the best prediction for PE and *Chla* was the PLSR model with spectra preprocessed by S-G smoothing, followed by standardization calculation. The optimal method for PC and APC prediction was the SVR model with S-G smoothing followed by SNV preprocessing.

### Validation of the optimal prediction models

On the basis of the optimal models established above, 24 field-collected *N. yezoensis* samples were used for validation. Among the 4 pigments, PE content was still predicted best by the PLSR model combining S-G + standardization with *R*_W_^2^, RMSE, and MAPE of 0.9481, 0.3507 mg/g, and 14.64%, respectively. The residuals of the PE prediction ranged from −0.5063 to 0.8265 mg/g. For PC and *Chla*, the prediction *R*_W_^2^ was also greater than 0.9, and MAPE was less than 15%, indicating high prediction accuracy. The prediction of APC was still unsatisfactory, with *R*_W_^2^ of less than 0.9 and MAPE significantly higher than 20%. The results showed that the optimal models established in this study could be used for the quantitative phenotyping of PE, PC, and *Chla* content in field samples of *N. yezoensis*.

### Prediction maps of phycobiliproteins and *Chla* in *N. yezoensis*

The visualized maps representing phycobiliproteins and *Chla* content predicted by the best models are shown in Fig. 5, along with an RGB image of the *N. yezoensis* thalli. The pigment content is presented in terms of the gradient of color variation from blue to red. The content gradually increased from the holdfast to the tip, through the entire thalli for all 4 pigments. From these visualized images, through our established models, the pigment contents could be deduced from any point on the thalli rather than from the average values of the whole thalli as per traditional methods.

## Discussion

### HSI for high-throughput phenotyping

**Fig. 4.**
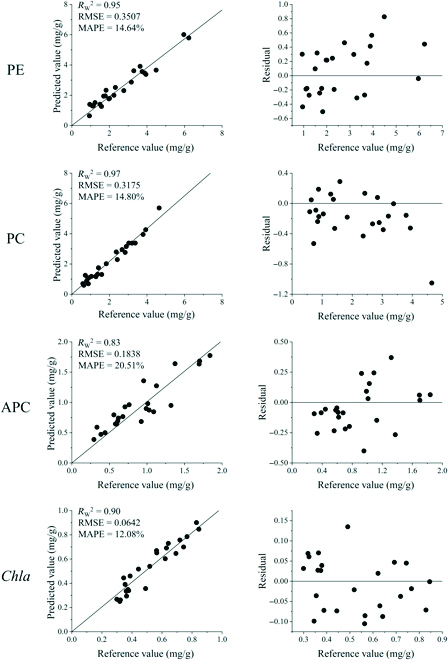
Scatter diagrams and residual analysis plots of reference value vs. the predicted value of PE, PC, APC, and *Chla* contents in field samples using the PLSR and SVR models.

Monitoring the physicochemical properties of plants and their growth while interacting with the surrounding environment is one of the most important aspects of plant phenotyping [61,62]. However, high-throughput phenotyping methods for investigating the phenotypes of macroalgae are still in the early stages of development [39]. Wet chemistry methods to evaluate phycobiliprotein and chlorophyll contents are time-consuming, laborious, destructive, and highly experience-dependent. Usually, it takes about 4 h to extract phycobiliproteins and chlorophyll from the thalli of *Neopyropia* using the traditional method. However, using HSI, it took only 2 to 3 min to spread the *N. yezoensis* thalli to avoid the overlapping influence on reflectance and another approximately 2 min for image acquisition using a hyperspectral camera in a nondestructive manner. Overall, we estimated that the HSI approach for predicting phycobiliproteins and chlorophyll contents was at least 50 times faster than traditional methods in terms of data collection. In addition, with the standard methods established in this study, the optimal prediction models showed satisfactory robustness and accuracy for both laboratory and field samples (Figs. 3 and 4).

Moreover, on the basis of HSI, it is possible to monitor the thallus over its lifetime and on a large scale. Once the traits can be measured accurately across large numbers of individual thalli, specific regions of the genome or genes controlling trait variations can be identified using quantitative genetic tools [34]. Compared with traditional destructive methods, multiple properties of any point of interest can be modeled and assessed simultaneously from one image using HSI, which could further improve the throughput and reduce the cost of the measurement [28]. Furthermore, this fast and nondestructive phenotyping method could also be used on other economical macroalgae thalli such as *Neoporphyra haitanensis*, *Undaria pinnatifida*, and *Saccharina japonica*. The hyperspectral data collection process is faster and more convenient for most macroalgae.

As one of the most important macronutrients, N forms parts of proteins, free amino acids, and chlorophyll molecules [63]. In *N. yezoensis* thalli, the phycobiliproteins and *Chla* contribute approximately 40% of cell proteins [5], which could be used to assess N content. In this study, the prediction model of phycobiliproteins and *Chla* content using HSI system detecting wavelengths of 400 to 1000 nm could be extended to the evaluation of N content in *N. yezoensis*, which usually strongly correlates with wavelengths of 1,300 to 2,500 nm in higher plant [64,65].

### HSI on nondestructive prediction of phycobiliproteins and *Chla* contents

**Fig. 5.**
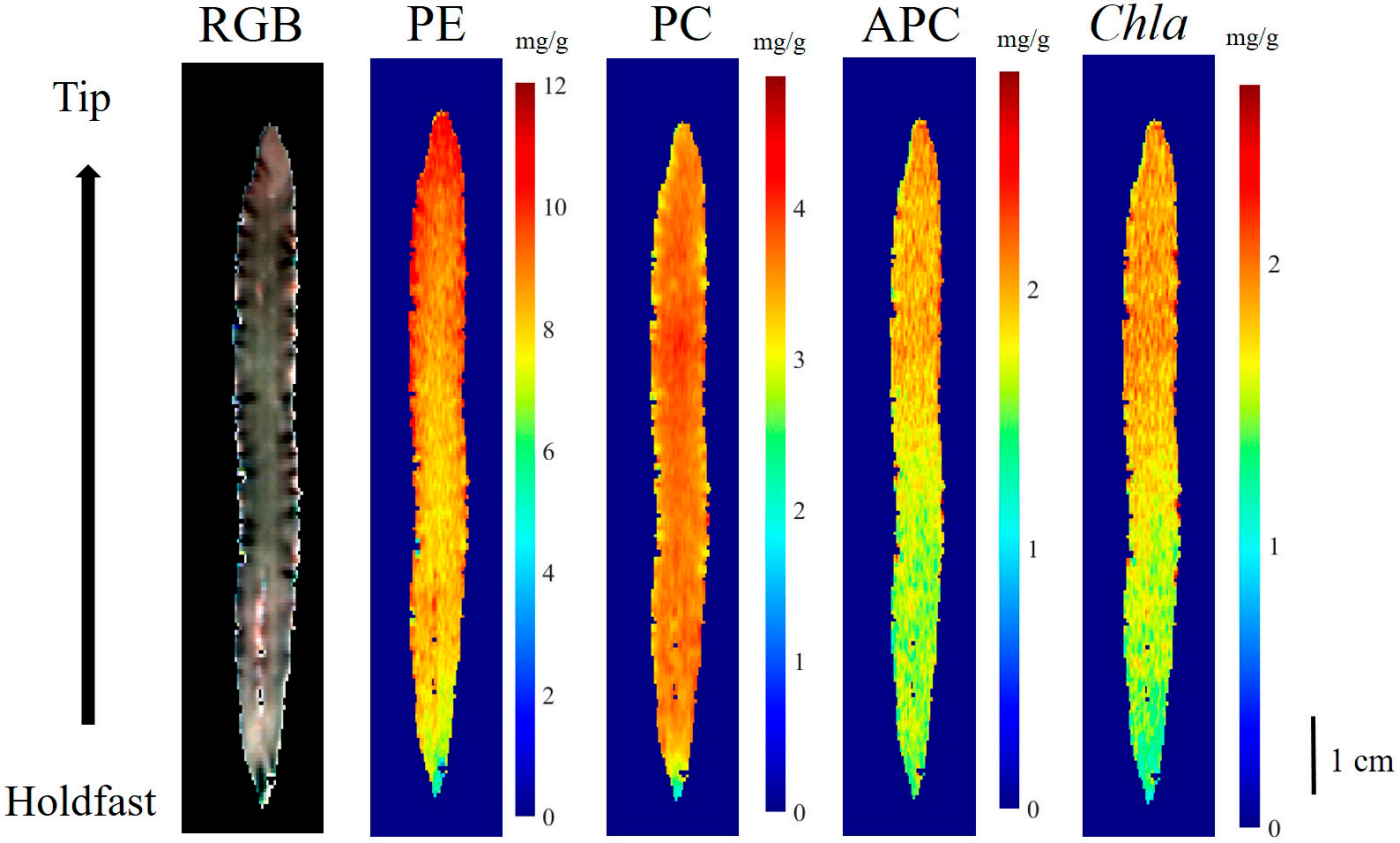
RGB image and prediction maps of PE, PC, APC, and *Chla* content in *N. yezoensis* thalli.

HSI, which combines spatial information and high-resolution spectral reflectance data, is a promising and noninvasive method for detecting biochemical variations in plants [31,66]. This study introduced the HSI system to predict phycobiliprotein and chlorophyll content in *Neopyropia*. On the basis of multivariate modeling analysis, the established models could be used for the quantitative prediction for PE, PC, and *Chla* with high accuracy (Tables 2 and 3). Using the HSI system not only provides numerical information for pigment contents of the whole thalli rapidly and nondestructively but also visualizes the distribution maps of pigment content through a thallus. The spatial distribution of PE, PC, APC, and *Chla* observed for *Neopyropia* showed no homogeneity throughout the individual thallus, and values of any point of interest on the thallus could be deduced from our models (Fig. 5). In addition, lower pigment concentrations were observed near the holdfast than in other parts, corroborating that the cell base region near the holdfast was the region of rapid cell division in *N. yezoensis*. Similarly, in Laminariales, a lower pigment content was found in meristematic cells at the bottom of the blade than in other parts of the blade cells [67]. Overall, the prediction maps of the 4 pigments provided a visual representation of spatial heterogeneity within the thallus of *N. yezoensis*. Using the HSI system, this study is the first successful estimation of phycobiliprotein and chlorophyll content in red algae.

The stress response in algae is complex and involves a variety of physiological and biochemical adaptions at different levels, leading to pigment changes and reallocation [Asaari et al., 2018; 68]. The composition of PE and PC vary significantly during chromatic adaptation [5]. The elimination of nitrogen from the growth medium of red algae leads to the rapid degradation of PE, followed by the degradation of the PC hexamer [49]. In this study, *Neopyropia* thalli responded to various light qualities and nutrition conditions by producing different concentrations and proportions of pigments, which supplied adequate range data for modeling in the HSI system. The variation in pigment content can also be used to evaluate different responses and adaptive capacities under different stress conditions [69]. It is possible to detect genotypic differences among different strains by nondestructively observing the response of physiological and biochemical phenotypes to various stresses using the HSI system.

### Advantages of multivariate modeling analysis based on full spectra for HSI

The spectral characteristics of *N. yezoensis* were different from those of higher plants. Absorption bands at approximately 500 and 570 nm were due to the unique absorption of PE [5]; meanwhile, a salient absorption near 620 nm should be attributed to the presence of PC [70]. Moreover, absorption features appeared near 440 and 660 nm owing to chlorophylls [71,72]. The absorption at approximately 970 nm may be related to the O–H stretching vibration [60,73]. Thalli with low-pigment concentrations exhibit relatively high reflectance values throughout the entire spectral region [69]. These differences revealed that the physicochemical properties of the thalli were altered during the experimental treatments [74].

The potential of hyperspectral sensing has not been fully realized in previous studies, and it commonly uses a simple mode of vegetation indices derived from only 2 or 3 bands [75]. Vahtmäe et al. [45] used 9 vegetation indices to predict chlorophyll-a, chlorophyll-b, and carotenoid concentrations in the marine macroalgae *F. vesiculosus*, *C. glomerata*, *Chara aspera*, and *Chara horrida*. The result showed that the coefficient of determination (*R*^2^) between chlorophyll-a, chlorophyll-b, and vegetation indices were between 0.41 and 0.67 depending on the index within 4 macroalgae. Choo et al. [76] used normalized difference vegetation index to predict chlorophyll content with a correlation coefficient of 0.70. However, vegetation indices only consist of a minimal number of spectral bands, which might ignore the characteristic bands of traits [68], and the indices might not be suitable for diverse species [6]. Moreover, all complex compounds (such as pigments, lipids, and carbohydrates) contribute to the spectral characteristics of the blade [28]; therefore, specific compounds might not be predicted accurately using vegetation indices.

In this study, multivariate modeling analysis based on full spectra was used to process image data to establish more accurate models of vegetation indices for the photosynthetic pigment contents in *N. yezoensis*. The *R*_Train_^2^, *R*_Test_^2^, and *R*_W_^2^ of optimal models for PE, PC, and *Chla* concentrations predictions were all greater than 0.9, and MAPE of the 3 pigments were all less than 15%, that meaning they could be used for quantitative analysis with satisfactory accuracy and robustness (Tables 2 and 3 and Figs. 3 and 4). The PE prediction model showed the best performance with a prediction error of 0.4804 mg/g in the range of 1.840 to 13.450 mg/g. The prediction error of the PC and *Chla* content was 0.3068 mg/g in the range of 0.960 to 5.270 mg/g and 0.1530 mg/g in the range of 0.290 to 2.230 mg/g, respectively. For APC content, the model could only be used for qualitative analysis with the best *R*_Test_^2^ and RPD values of 0.8399 and 2.53, respectively, with a prediction error of 0.2232 mg/g in the 0.430 to 2.350 mg/g range. This may be attributed to the small variation in the APC content under the experimental conditions [77] and the overlap of the absorption peak between APC (650 nm) and *Chla* (660 nm). Multivariate modeling analysis plays an essential role in extracting meaningful information from wide-range spectral data for qualitative and quantitative analyses [57].

In our study, the SVR model performed better than the PLSR model, indicating a more precise prediction. Mountrakis et al. [56] also reported that SVR often produces higher accuracy than traditional methods because of its ability to handle small training datasets successfully. The SVR models performed better than the PLSR models in the quantitative determination of biochemicals, such as phosphorus in seafood [57], soluble solid content in sweet potato [78], and soluble solid content in *Agaricus bisporus* [79]. Ge et al. [28] used Vis–NIR–SWIR to determine leaf physiological traits in maize, and the SVR model performed slightly better than the PLSR model for 5 traits.

Although the optimal models showed satisfactory results, the model performed better on the laboratory samples than on the field-collected samples. The results might be attributed to the range of pigment content in the laboratory samples that could not cover that of the field-collected samples. It has been found that content outside the modeling range may lead to extra prediction errors [80]. In addition, other machine learning approaches such as random forest, convolutional neural network, artificial neural network, and deep learning methods could also be considered for a robustness test in future work. Moreover, the genetic variations between laboratory and field-collected samples may increase the complexity of the analysis [63,81]. Genetics and breeding with high-throughput phenotyping generally involve hundreds of genotypes; therefore, more *N. yezoensis* genotypes should be used to improve the application of the model in further studies.

## Data Availability

The data used to support the findings of this study are available from the corresponding author upon reasonable request.
